# Electro-Thermal Co-Design and Verification of TGV Transmission Structures for High-Power High-Frequency Applications

**DOI:** 10.3390/mi17020253

**Published:** 2026-02-16

**Authors:** Luming Chen, Zhilin Wei, Shenglin Ma, Yan Chen, Yihan Xie, Chunlei Li, Shuwei He, Hai Yuan

**Affiliations:** 1 Pen-Tung Sah Institute of Micro-Nano Science and Technology, Xiamen University, Xiamen 361005, China; 19920210156229@stu.xmu.edu.cn (L.C.); 19920231151660@stu.xmu.edu.cn (Z.W.); 19920231151662@stu.xmu.edu.cn (Y.X.); lichunl2001@stu.xmu.edu.cn (C.L.); 2 School of Electronic Science and Engineering, Xiamen University, Xiamen 361005, China; chenyan0611@stu.xmu.edu.cn; 3 Chengdu Ganide Technology Co., Ltd., Chengdu 610000, China; heshw@ganide.com; 4 Xi’an Microelectronic Technology, Xi’an 710071, China; yhlk2005@163.com

**Keywords:** Through Glass Via, electro-thermal coupling, high-power, thermal resistance, continuous/pulsed wave

## Abstract

Through Glass Via (TGV) technology has emerged as a promising solution for advanced packaging. While glass offers lower dielectric loss than silicon, its lower thermal conductivity raises concerns about electro-thermal coupling effects in high-power, high-frequency applications. Therefore, this study conducted an electro-thermal co-design of TGV grounded Coplanar Waveguide (CPW) and Radio Frequency (RF) TGV connected CPW structures. A high-power test platform was developed to investigate the electrical and thermal performance of these structures. The temperature distribution mechanism under high-power conditions was revealed. Under high power and high frequency, the decrease in surface conductivity affected by surface state and film layer composition leads to increased loss, triggering temperature rise and forming an electrothermal coupling loop. Under continuous wave operation (5–20 W), the temperature rise reaches 92.4 °C while insertion loss increases by only 0.4 dB. Under pulsed wave operation (25–100 W, 2.5% duty cycle), the temperature rise is merely 2.1 °C with insertion loss increasing by 0.3 dB. The quadruple-redundant design and reduces heat flux density, preventing localized hotspot formation. The pulse intervals suppress thermal accumulation, leading to lower temperature rise. Therefore, continuous wave applications should prioritize thermal management, while pulsed wave applications can focus on electrical performance optimization.

## 1. Introduction

The rapid advancement of radar technologies, artificial intelligence, and autonomous driving has necessitated the development of modern RF systems capable of operating at higher frequencies with broader bandwidths, enhanced power handling capabilities, and increased integration density [1,2,3,4,5,6,7,8,9,10,11]. Within this technological landscape, advanced interposer technology has emerged as a critical enabling solution to address these stringent requirements. These interposers must exhibit superior electrical and thermal characteristics, including minimal insertion loss and a low coefficient of thermal expansion, while facilitating high-precision, high-density interconnect architectures to ensure optimal performance and reliability of RF systems [12,13]. Through Silicon Via (TSV) interposers, despite their advantages, present complex fabrication processes and substantial manufacturing costs [4,5,14,15,16,17]. LTCC (Low Temperature Co-Fired Ceramics) interposers are constrained by inherent challenges including co-firing shrinkage and elevated manufacturing costs. While Redistribution Layer (RDL) interposers offer superior routing precision, they suffer from relatively high dielectric losses and insufficient thermal dissipation capabilities, accompanied by significant substrate warpage issues. In recent years, TGV interposers have emerged as a promising solution for Radio Frequency System in Package (RF-SiP) modules, attributed to their low dielectric loss, tunable coefficient of thermal expansion (CTE), and superior dimensional stability [14,18]. Notably, TGV based coplanar waveguides fabricated by our research group in 2019 demonstrated a remarkably low insertion loss of 0.13 dB/mm at 10 GHz [19]. In 2021, Yu et al. achieved insertion loss of 0.22 dB at 80 GHz for TGV based coplanar waveguides [5,20]. Concurrently, Galler et al. developed wafer level RF module based on TGV interposer technology, achieving an insertion loss of 2.85 dB and antenna gain of 28 dBi at 162 GHz [14]. As RF systems evolve toward high-power, high-frequency operation, self-heating and electro-thermal coupling effects elevate device operating temperatures, inducing variations in material properties including dielectric constant, loss tangent, and electrical conductivity, which ultimately compromise transmission performance. This phenomenon has attracted considerable research attention in recent years.

In 2013, Roda et al. found that silicon based coplanar waveguides show significant performance degradation above 120 °C, with 0.2 dB/mm additional loss at 10 GHz [21]. Subsequently, Lee et al. investigated the effects of temperature changes from 25 °C to 100 °C on TSV channel S_21_ parameters, revealing an obvious degradation in S_21_ parameters as temperature rise [22]. These studies primarily focused on the impact of external thermal environments on the electrical performance of transmission structures. However, the self-heating effects induced by high-power signals and their degradation mechanisms on electrical properties remain unexplored.

In 2020, Min et al. established an equivalent thermal resistance and an electrical circuit network for coaxial-like TSV structures, and found that the significant change in the S_21_ parameters occurring to MOS capacitance’s is dependent on the temperature [23]. Tai et al. present a fabricated SiON thin-film based coplanar waveguides, achieving a loss of 2.92 dB/mm at 50 GHz and a power handling capability (APHC) of 10 W at 20 GHz [24]. Lu et al. developed a domain decomposition-based electro-thermal coupling simulation methodology for accurate prediction S_21_ parameter of TSV, revealing a 26% increase in insertion loss due to electro-thermal coupling effects at 2 GHz and 353 K [25]. Zaghari et al. adopted thermo-mechanical-electrical co-design methodologies for TSV and TGV optimization studies in 2025. Multi-objective optimization with machine learning approaches were implemented to analyze TSV/TGV performance characteristics, in terms of copper protrusion, thermal resistance, and electrical parasitic capacitance of TSV/TGV [26,27]. Son et al. conducted a thermal analysis of high bandwidth memory (HBM)-GPU modules considering power consumption. The study investigated the self-heating issue of HBM PHY [28]. Chien et al. evaluated the effect of temperature on the signal integrity of HBM links using the Peak Distortion Analysis method [29].

However, these works primarily remain at the theoretical level. Based on our literature survey, no existing research has demonstrated the actual implementation of a test vehicle under high-frequency high-power conditions. While prior theoretical investigations have contributed to understanding electrothermal coupling mechanisms, a gap persists between theoretical frameworks and physical design implementation.

The present work features two key innovations:

(1) This work designs and fabricates a high-frequency high-power transmission structure through optimized ground via layout and quadruple-redundant RF TGV signal structure design, which reduces local current density peaks and heat flux density peaks, achieving reductions in loss, thermal resistance, and temperature rise. Experimental validation demonstrates that the optimized structure exhibits reliable high-power transmission capability under both 20 W continuous wave and 100 W pulsed wave conditions.
(2) Based on the analysis and correlations between experimental and simulation results, we confirmed the electrothermal coupling mechanism whereby surface conductivity degradation induces increased loss and temperature rise as the designed TGV RF transmission structures transmit high power high frequency signals. Meanwhile, a simulation scheme considering electrothermal coupling effects was established as a design methodology to implement the design of high-power high-frequency TGV based RF transmission structures.

This study is divided into five Chapters. Section 2 introduces the electro-thermal co-design of TGV based transmission structures. Section 3 describes the fabrication process. Section 4 “Materials and Methods” presents three testing approaches, Section 4.1 for small-signal electrical performance testing (1 mW), Section 4.2 for large-signal electro-thermal coupling testing (10 W), and Section 4.3 for large-signal continuous wave and pulsed wave testing (20 W/100 W). Section 5 “Results and Discussion” corresponds to Section 4, where Section 5.1, Section 5.2 and Section 5.4 align with the testing methods in Section 4.1, Section 4.2 and Section 4.3, respectively, presenting experimental results under different power conditions. Additionally, Section 5.2 analyzes the electro-thermal coupling simulation method, while Section 5.3 provides theoretical analysis of thermal resistance and temperature rise in the transmission structure. Finally, the research results are compared with the current, international state of the art.

## 2. Electro-Thermal Co-Design of TGV Based Transmission Structures for High-Power High-Frequency Applications

### 2.1. TGV Electrically Grounded CPW Structure Design

**Figure 1:**
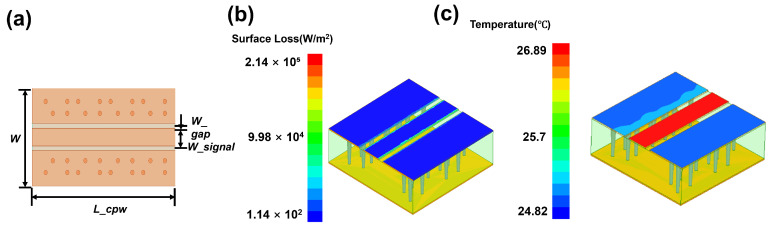
(**a**) Conceptual design diagram of CPW Structure simulated (800 μm); (**b**) heat flux density distribution of CPW structures under 1 W@18 GHz conditions (800 μm); (**c**) temperature rise distribution of CPW structures under 1 W@18 GHz conditions (800 μm).

**Figure 2:**
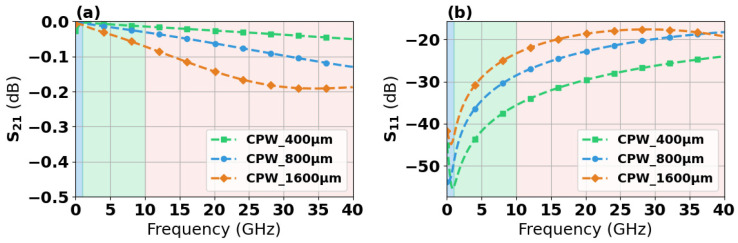
Simulated S-parameters versus frequency for CPW structures with lengths of 400 μm, 800 μm, and 1600 μm. S21 (**a**), S11 (**b**).

This study designed CPW structures featuring a signal line width of 150 μm, varying lengths of 400 μm, 800 μm, and 1600 μm, and a characteristic impedance of 50 Ω. We optimized the signal line width through parametric analysis. The technical concept diagram is shown in Figure 1a, and the structural dimensions are listed in Table 1. Electrical analysis was conducted using Ansys High Frequency Structure Simulator (HFSS). Figure 2 demonstrates that insertion loss increases with transmission line length. Figure 1b,c present simulation analyses of surface heat flux density and temperature rise characteristics for the structures using HFSS and ICEPAK. The results in Figure 1c indicate that under 1 W@18 GHz input power conditions, the temperature distribution of the CPW is uniform along the transmission direction. In addition, the heat flux density distribution and temperature rise characteristics of CPW structures with lengths of 400 μm and 1600 μm were also investigated. The detailed results are presented in Figures S1 and S2 in the Supplementary Materials. In the following thermal simulation, the side surfaces were set as adiabatic boundaries, and the bottom surface was set as a constant temperature boundary at 25 °C.

### 2.2. RF TGV Connected CPW Structure Design

**Figure 3:**
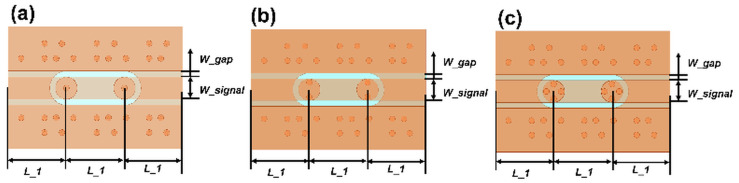
Top view of RF TGV connected CPWs with different configurations: (**a**) single TGV, (**b**) dual-redundant TGV, and (**c**) quad-redundant TGV structure.

**Figure 4:**
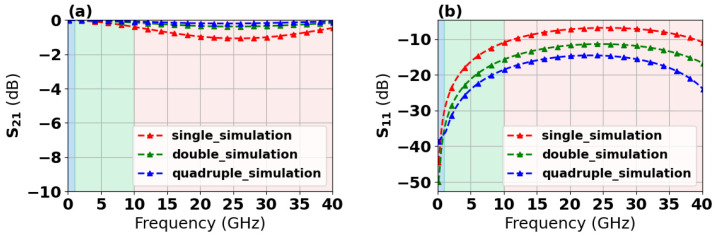
Simulated S-parameters versus frequency of single TGV, dual-redundant TGV, and quad-redundant TGV transmission structures. S21 (**a**), S11 (**b**).

**Figure 5:**
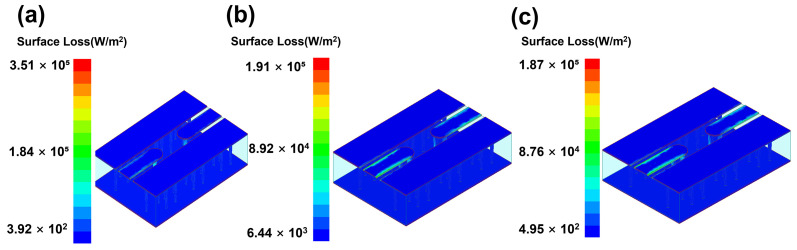
Surface heat flux density distribution of RF TGV connected CPW structures with different configurations: (**a**) single TGV, (**b**) dual-redundant TGV, and (**c**) quad-redundant TGV under 1 W@18 GHz conditions.

**Figure 6:**
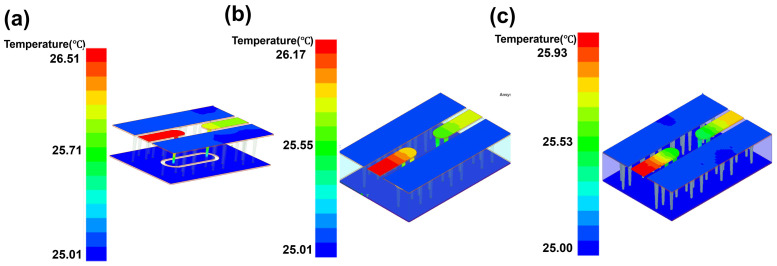
Temperature distribution of RF TGV connected CPWs with different configurations: (**a**) single TGV, (**b**) dual-redundant TGV, and (**c**) quad-redundant TGV transmission structures under 1 W@18 GHz conditions.

With the designed TGV electrically grounded CPW, three types of RF TGV connected CPW structures were designed with single, dual-redundant and quad-redundant RF TGV configurations. Figure 3 presents the technical concept diagram. Figure S3 presents the simulation models. The S-parameters were obtained through simulation analysis in the DC-40 GHz frequency range as shown in Figure 4. Compared with the designed CPW shown in Figure 2, RF TGVs cause an additional insertion loss of 0.3 dB at 18 GHz. With redundant RF TGV configuration, it is helpful to reduce transmission loss that can be attributed to the rise in the effective signal TGV diameter due to the larger number of RF signal TGVs [30,31]. Meanwhile, the quad-redundant structure exhibits significantly lower return loss compared to other TGV transmission structures. Figure 5 illustrates the maximum heat flux density distribution for single, dual-redundant, and quad-redundant RF TGV configurations. The maximum heat flux density is located at the edges of the signal lines of CPW and the sidewalls of TGVs, which is similar to the designed CPW. But an obvious rise of 55% in the heat flux density can be found, which is in accordance with the increasing insertion loss with RF TGVs. Compared to single TGV configurations, quad-redundant TGVs exhibit a notable reduction in maximum heat dissipation power density, decreasing from 3.51 × 10^5^ W/m^2^ to 1.7 × 10^5^ W/m^2^, a reduction of approximately 50%. Figure 6 presents the temperature distribution of TGV structures. As the number of RF signal redundant TGV increases, the maximum temperature rise also shows a decreasing trend. It can be observed that redundant RF TGV configurations help reduce equivalent thermal resistance and enhance heat dissipation capability. Additionally, the highest temperature is found to occur at the CPW signal lines. As shown in Figure 6, the RF TGV connected CPW structure exhibits higher temperatures at the input port than at the output port, with a difference of approximately 0.8 °C, which is different with designed CPW structures. We think that it can be ascribed to the decline in the effective input signal power and dissipating heat as RF signal goes through forward, which will be discussed in Section 5.

## 3. Fabrication TGV Based RF Transmission Structures

**Figure 7:**
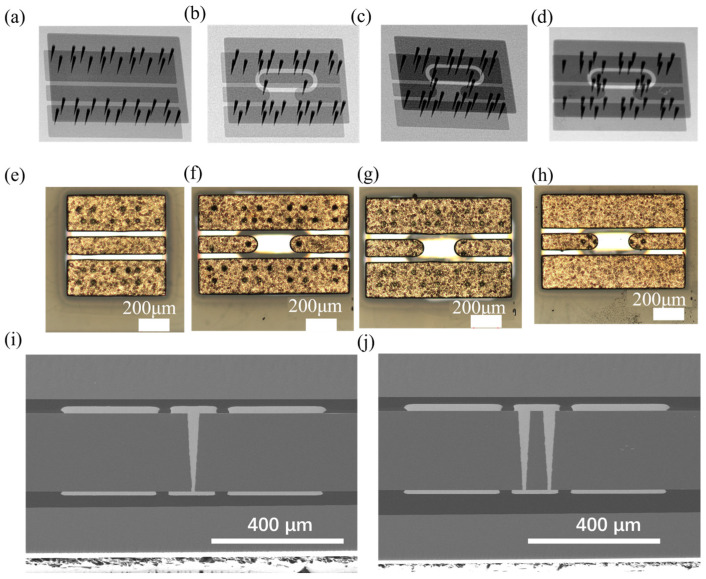
(**a**) X-ray image of CPW, (**b**) X-ray image of single RF TGV connected CPWs, (**c**) X-ray image of dual-redundant RF TGV connected CPWs, (**d**) X-ray image of quad-redundant RF TGV connected CPWs, (**e**) optical microscope image of CPWs (top view), (**f**) optical microscope image of single RF TGV connected CPWs (top view), (**g**) optical microscope image of dual-redundant RF TGV connected CPWs (top view), (**h**) optical microscope image of quad-redundant RF TGV connected CPWs (top view), (**i**) SEM cross-sectional image of single RF TGV connected CPWs, and (**j**) SEM cross-sectional image of quad-redundant RF TGV connected CPWs.

Blind TGVs are formed on 8-inch BF33 glass wafers using LIDE technology, followed with a barrier and seed layer of Ti/Cu deposition by PVD. TGVs are filled solidly by the Cu electroplating process; the CMP process is employed to remove seed layer and barrier layer sequentially. Cu rewiring layer is fabricated using the SAP process and protected with a PI passivation layer. Then, it is bonded to a carrier wafer with temporary bonding process, its backside is subjected to grinding and CMP processing to expose the Cu TGV terminals, followed with backside Cu/PI RDL fabrication. Electroless nickel–palladium–gold plating is applied for future wire bonding process. Finally, debonding and dicing are performed. The samples are shown in Figure 7.

## 4. Materials and Methods

### 4.1. Electrical Performance Testing Method Under Small-Signal Conditions (1 mW)

The small-signal (1 mW) electrical performance of TGV-based transmission structures was characterized over the 0–50 GHz frequency range. The measurement setup comprised a KEYSIGHT PNA-X vector network analyzer (Keysight Technologies, Santa Rosa, CA, USA) and Cascade Microtech GSG probes (Cascade Microtech, Beaverton, OR, USA) with a 150 μm pitch. Prior to measurement, the system was calibrated using an Anritsu 36804B-10M calibration kit (Anritsu Company, Morgan Hill, CA, USA) following the standard SOLT procedure.

### 4.2. Electro-Thermal Coupling Performance Testing Method Under Large-Signal Conditions (10 W)

**Figure 8:**
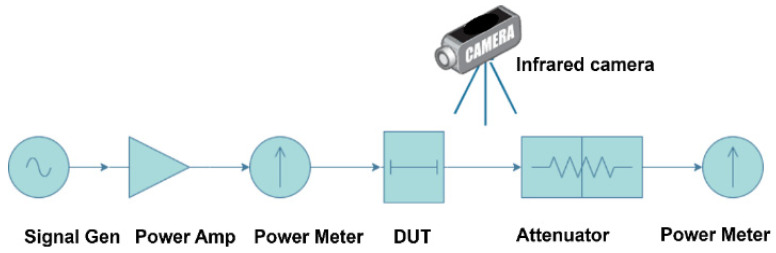
Test platform for electro-thermal characteristics of transmission structures under high-power RF signal conditions.

Considering the practical application requirements and measurement conditions, RF signal input of 10 W@6 GHz, 7.9 W@12 GHz, and 6.3 W@18 GHz. The measurement method consists of the following steps. First, in the test platform shown in Figure 8, the TGV transmission structure is configured in a short-circuit condition, the probe loss and cable loss are tested and summarized in Table 2. Next, the TGV transmission structure is connected electrically normally, the total insertion loss including the probe, cable, and TGV transmission structure is obtained. Finally, the insertion loss of TGV transmission structure can be derived. Throughout the measurement process, infrared thermal imaging is utilized to acquire the temperature rise distribution across the test structure surfaces. The insertion loss and temperature rise distribution of the samples transmitting a high-power RF signal is measured by the test platform. Figure 8 shows the test platform, which consists of the following equipment, an Anritsu MG3694C RF signal generator Anritsu Company, CA, USA), a Ceyear 3871EB power amplifier (Ceyear Technologies Co., Ltd., Qingdao, China), a Keysight N1912A power meter (Keysight Technologies, CA, USA) with a measurement accuracy of ± 0.05 dB, Cascade Microtech GSG probes with a 150 μm pitch (Cascade Microtech, OR, USA), and a FLIR A615 infrared thermal imaging camera (FLIR Systems, Wilsonville, OR, USA) with a temperature measurement accuracy of ±0.5 °C.

### 4.3. Electro-Thermal Performance Characterization Method Under Large-Signal Continuous Wave (20 W) and Pulsed Wave Conditions (100 W)

**Figure 9:**
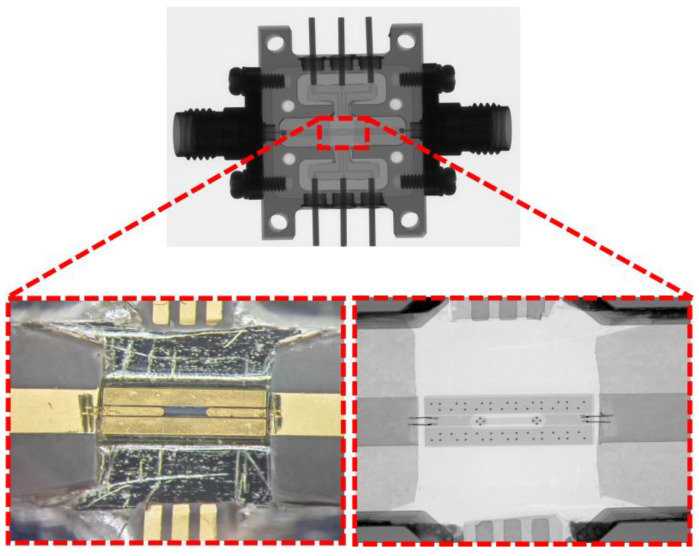
X-ray image of the test structure after mounting, with the RF TGV connected CPW structure highlighted within the red dashed box.

**Figure 10:**
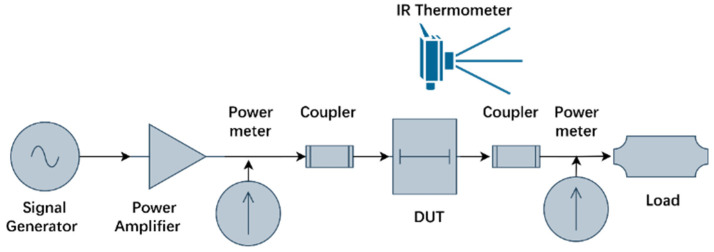
Test platform for electro-thermal characteristics of transmission structures under continuous wave (20 W) and pulsed wave conditions (100 W).

To validate the electrical-thermal co-design benefits, the quad-redundant RF TGV-connected CPW structure was mounted in a customized aluminum alloy fixture to mimic the application scenario. Two 50 Ω microstrip transmission lines were positioned at the input and output sides. The electrical connections were established through wire bonding, as illustrated in Figure 9. With this configuration, the input continuous wave power was able to scale up to 30 W while the input pulsed power was able to scale up to 120 W. A test platform was established as shown in Figure 10, where signals are generated by a signal generator and sequentially pass through a power amplifier, flexible waveguide, circulator, rigid waveguide, coupler, and waveguide-to-coaxial adapter to reach the quad-redundant TGV structure. Output signals then pass through a waveguide-to-coaxial adapter, rigid waveguide, and coupler before connecting to the load. The insertion loss of the S_21_ was measured using a method similar to that described in Section 4.2. First, at 20 GHz, power meters were employed at both ends to measure the input and output power, from which the total transmission loss was obtained by calculating their difference. Subsequently, the cable line loss of 3.1 dBm and the fixture loss of 0.8 dBm were subtracted from the total loss to determine the actual insertion loss of the TGV transmission structure. It should be noted that the insertion loss of the gold wire bonding is neglected in this analysis due to its negligibly small value. Temperature monitoring of the device under test is achieved through an infrared thermometer connected to a computer.

## 5. Results and Discussions

### 5.1. Electrical Performance Characterization of TGV Based Transmission Structures Under Small Signal Conditions (1 mW)

**Figure 11:**
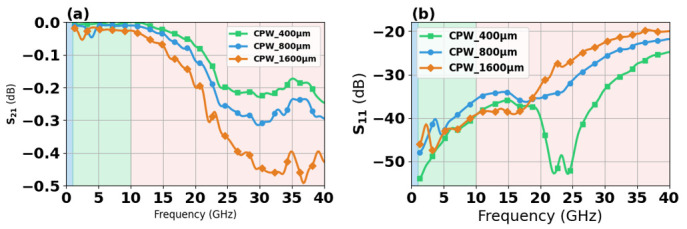
S-parameter frequency response of CPW (**a**) S21 (**b**) S11.

CPW transmission structures of 400 μm, 800 μm, and 1600 μm in length were characterized for S-parameter performance under small-signal conditions. As shown in Figure 11, all three transmissions exhibit a low loss less than 0.2 dB in range of DC-20 GHz. The transmission structure with a length of 1600 μm demonstrates an insertion loss of approximately 0.45 dB at 40 GHz. The S_11_ parameter reveals that all transmission structures exhibit minimal reflection in the DC-20 GHz range, approximately −40 dB to −50 dB. However, as the frequency increases to the 20–40 GHz range, the reflection coefficient stabilizes at approximately −20 dB.

**Figure 12:**
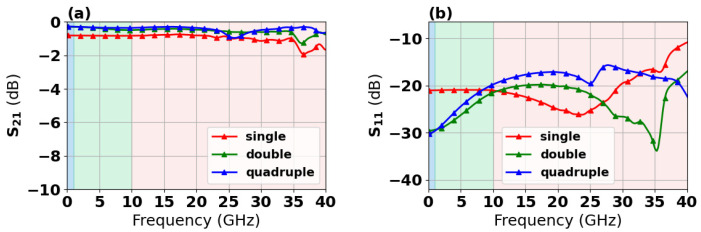
Measured S-parameters of single, dual-redundant, and quad-redundant RF TGV connected CPW structures: (**a**) S21 (**b**) S11.

S-parameters of single, dual-redundant, and quad-redundant RF TGV connected CPW structures were measured under small-signal excitation. Figure 12a demonstrates that quad-redundant RF TGV configurations exhibit lower transmission loss, approximately 0.2 to 0.5 dB in the range from DC to 40 GHz, while the single RF TGV structure shows higher transmission loss, approximately 0.8 to 1.5 dB. Figure 12b shows that all three structures maintain return loss below 15 dB in the range from DC to 20 GHz, validating a good impedance matching. The study reveals that the quadruple-redundant structures exhibit the lowest insertion loss. Although TGVs introduce additional parasitic capacitance and inductance, the adverse effects of these parasitic parameters on overall transmission performance can be effectively suppressed through rational optimization of TGV spacing and spatial layout. The quadruple-redundant structures possess a larger equivalent cross-sectional area, which promotes more uniform high-frequency electromagnetic field distribution and effectively reduces localized high-loss regions caused by field concentration effects, thereby lowering overall energy dissipation. Therefore, with parasitic effects adequately suppressed, the quadruple-redundant structures achieve the minimum insertion loss.

The L-2L de-embedding method was employed to extract the insertion loss of single, dual-redundant, and quad-redundant RF TGV at 18 GHz [27]. The results indicate that the insertion loss of the single TGV is 0.37 dB@18 GHz, the double TGVs 0.23 dB@18 GHz, and the quadruple TGV structure is 0.12 dB@18 GHz. The data demonstrates that the insertion loss exhibits a decreasing trend with the increase in the number of TGVs. Figure S4 compares the simulated and measured S21 parameters of CPW, and RF TGV connected CPW structures at 18 GHz@ 1 mW. In general, the S21 parameter simulation and measurement maintain good consistency.

### 5.2. Electrical Performance Characterization of TGV Based Transmission Structures Under Large Signal Conditions (10 W)

**Figure 13:**
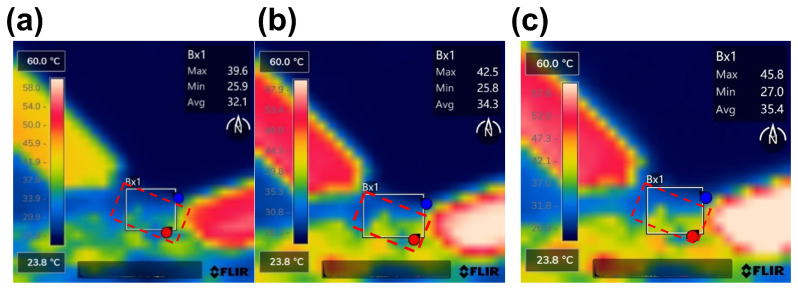
Measured temperature rise distribution contour maps of CPW structure: (**a**) 10 W@6 GHz, (**b**) 7.9 W@12 GHz, and (**c**) 6.3 W@18 GHz.

**Figure 14:**
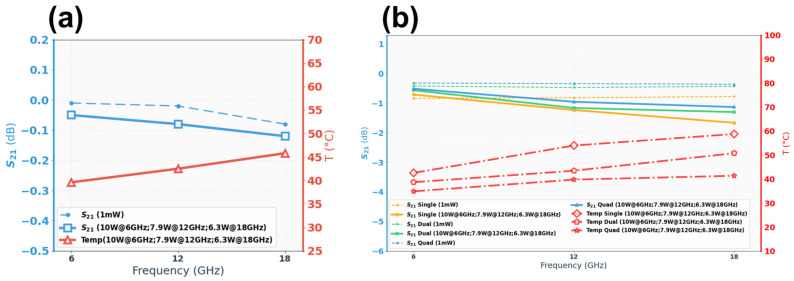
Measured insertion loss for small-signal (1 mW) and large-signal (6.3–10 W) excitations, and corresponding temperature rise as functions of frequency (**a**) CPW (**b**) RF TGV connected CPW structures.

Figure 13 shows the temperature rise distribution contour maps of the CPW structure under a large RF signal input of 10 W@6 GHz, 7.9 W@12 GHz, and 6.3 W@18 GHz, respectively. As shown in Figure 13, the red dashed box indicates the test structure location at the center between the two probes, while the white solid boxes denote the maximum and minimum temperature regions identified by the infrared software. The insertion loss and temperature rise for the CPW structure (800 μm) are summarized in Figure 14a. To better validate the benefits of the electro-thermal co-design, insertion loss measured with a small-signal input of 1 mW is also included. It can be found that the degradation of insertion loss is about 0.03 dB at 18 GHz, justifying that the proposed CPW structure maintains good electrical performance under high-power operating conditions.

**Figure 15:**
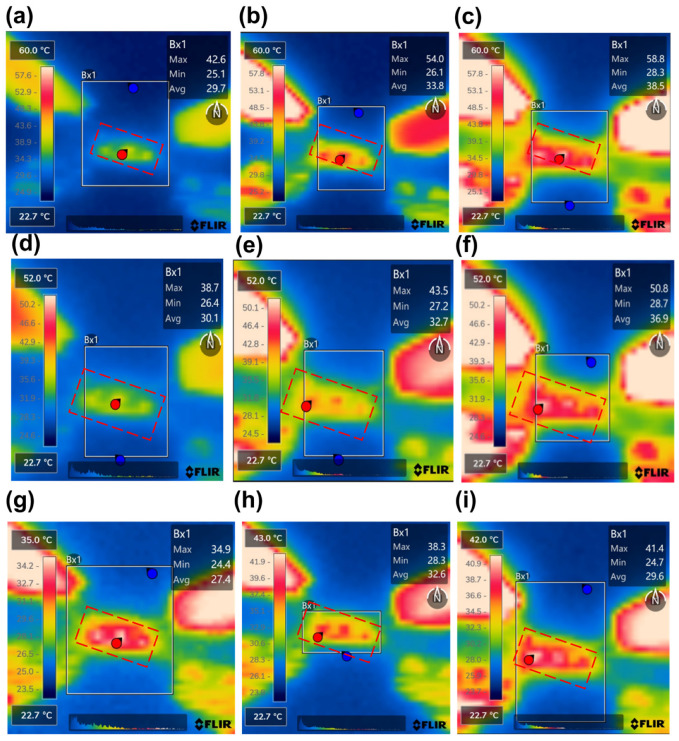
Measured temperature rise distribution contour maps of single redundant RF TGV connected CPW structure (**a**) 10 W@6 GHz (**b**) 7.9 W@12 GHz (**c**) 6.3 W@18 GHz; double redundant RF TGV connected CPW structure (**d**) 10 W@6 GHz (**e**) 7.9 W@12 GHz (**f**) 6.3 W@18 GHz; quadruple redundant RF TGV connected CPW structure (**g**) 10 W@6 GHz (**h**) 7.9 W@12 GHz (**i**) 6.3 W@18 GHz.

Figure 15 show the temperature rise distribution of the structures under a large RF signal input of 10 W @6 GHz, 7.9 W @12 GHz, and 6.3 W@18 GHz, respectively. Insertion loss and temperature rise for RF TGV connected CPW structures are summarized in Figure 14b. Among the three designs, quad-redundant configuration temperature rise was limited to 34.9–41.4 °C across the 6–18 GHz frequency range under 6.3–10 W power. Compared with that under small-signal conditions, the insertion loss under large-signal conditions shows obvious degradation. Relative to small-signal conditions, large-signal operation exhibits temperature rise and pronounced insertion loss degradation. This phenomenon stems from Joule heating generated during high-power signal transmission, which elevates the structural temperature. The resulting thermal increase further degrades the electrical conductivity of metallic conductors and the loss tangent of dielectric materials, consequently leading to enhanced insertion loss through electro-thermal coupling effects. The quad-redundant structure exhibits the lowest insertion loss degradation of 0.2 dB, 0.32 dB, and 0.78 dB at 6 GHz, 12 GHz, and 18 GHz, respectively. This demonstrates that increasing the number of redundant signal TGVs effectively mitigates insertion loss degradation due to the electro-thermal coupling effect. The above results demonstrate the effectiveness of the proposed electro-thermal co-design approach for the TGV transmission structure.

**Figure 16:**
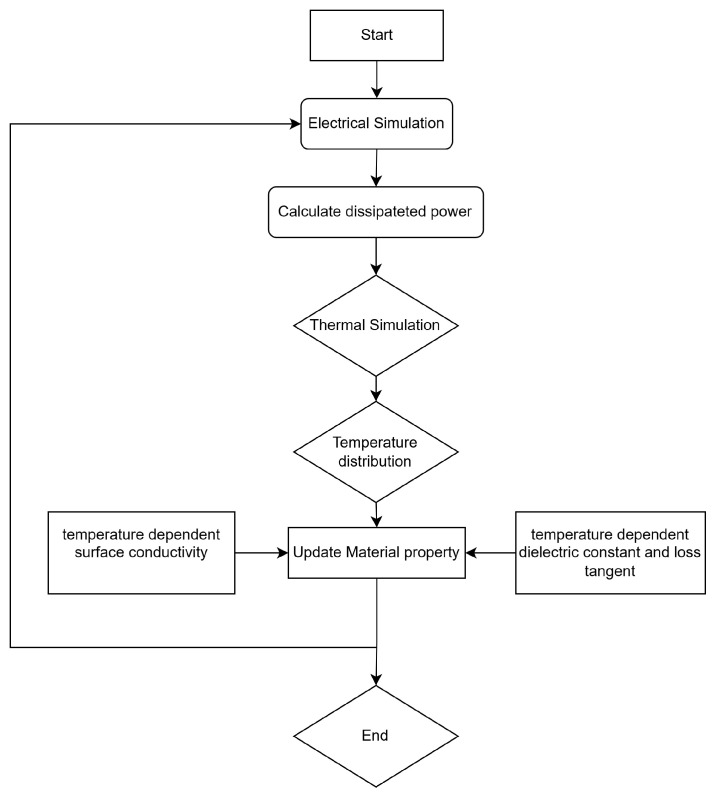
Workflow of electro-thermal coupling simulation.

To gain deeper insights into the insertion loss degradation test with large RF signal input, electro-thermal coupling simulations for TGV based transmission structure were carried out. Figure 16 shows the implementation process of the electro-thermal coupling simulations.

We conducted the electro-thermal coupling simulation using the HFSS and ICEPAK modules in Ansys Electronics. The governing equation for electro-magnetic field simulation of the transmission structure adopts the vector wave Equation (1)

(1) $$\nabla \cdot \left(\frac{1}{\mu_{r}}\nabla \times E\right)-k_{0}^{2}\varepsilon_{r}E=-jk_{0}Z_{0}J$$

where ε_r_ and μ_r_ are the relative dielectric constant and relative permeability respectively, *Z*_0_ is the characteristic impedance of free space, *J* is the excitation current source, *k*_0_ is the wave number of the field, and j is the imaginary unit.

The first-order absorbing boundary condition adopted is:

(2) $$\frac{1}{\mu_{r}}\hat{n}\times \left(\nabla \times E\right)+jk_{0}\sqrt{\frac{\varepsilon_{r}}{\mu_{r}}}\hat{n}\times \left(\hat{n}\times E\right)=0$$

The governing equation for thermal analysis is the Poisson equation:

(3) $$\nabla \cdot \left(k\nabla T\right)=-P$$

where *k* is the thermal conductivity, *T* is the temperature distribution, and *P* is the heat source [32].

Firstly, electromagnetic field simulation was performed in HFSS to obtain the power dissipation distribution of the CPW test structure.

**Figure 17:**
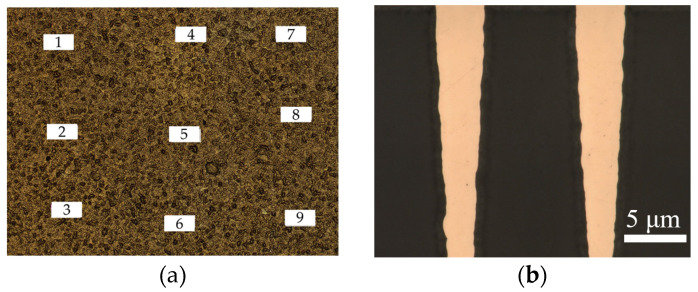
(**a**) Microscopic top view of CPW structure surfaces and (**b**) cross-sectional view of TGV sidewalls.

**Figure 18:**
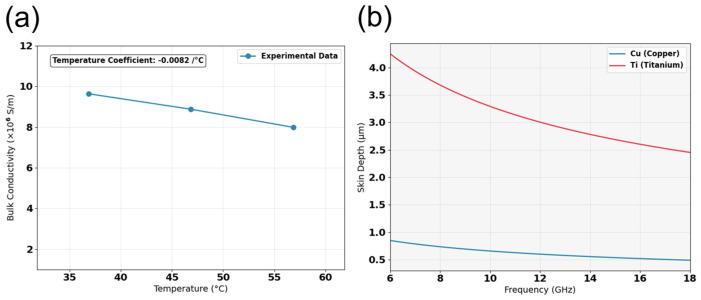
Measured bulk conductivity of copper versus temperature (**a**) Skin depth of Cu and Ti as a function of frequency (**b**).

**Figure 19:**
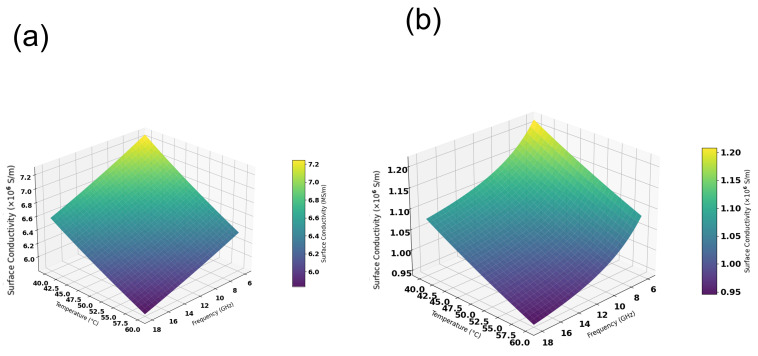
Surface conductivity of copper as a function of temperature and frequency (**a**) CPW (**b**) TGV.

In electro-thermal coupled simulations, the surface roughness and surface conductivity of metal are key input parameters that affect the simulation accuracy [33]. In this regard, we conducted the following studies. Figure 17a presents the surface micro-morphology of the transmission structure measured by a three-dimensional laser optical profiler. Through measurements at nine positions on the sample surface, the root mean square (RMS) value of surface roughness ranged from 0.5 μm to 0.8 μm, with a statistical average of 0.6 μm. Accordingly, 0.6 μm was adopted as the surface roughness input parameter in the electrothermal coupling simulations for the CPW structures. The surface morphology of TGV sidewalls was characterized as shown in Figure 17b, it can be found that TGV sidewalls exhibit considerably higher surface roughness than the planar region. We measured the bulk conductivity of the copper using a semiconductor device probe testing system. Figure 18a illustrates the temperature dependence of copper bulk conductivity. The results are lower than the theoretical value, which is primarily attributed to impurities introduced during the electroplating process. The measured temperature coefficient of resistance for the electroplated copper is 0.0082/°C, which exhibits a comparable order of magnitude and temperature-dependent characteristics of the theoretical value of pure copper 0.0043/°C. The frequency-dependent skin depth variation of Ti/Cu was also calculated. The Hammerstad model was employed in our simulations to account for the roughness-induced conductor losses. Rather than modeling the Ti barrier layer as a separate geometric entity, we employed an equivalent surface impedance approach where the Ti/Cu bilayer was simplified to a single-layer model with an effective surface conductivity. There is a Ti/Cu bilayer structure on the TGV sidewalls surface, which is different from Cu CPW structure. Given that the electrical conductivity of Ti is approximately 4% that of Cu, the presence of this titanium layer substantially reduces the effective conductivity of the structure. By calculating the effective conductivity based on the volume ratio of Ti/Cu layers within the skin depth and considering surface roughness, we applied Equation (4) [34] to determine the surface conductivity of the TGV sidewalls. As shown in Figure 19, the surface conductivity varied by approximately 17% across the temperature range of 40–60 °C and frequency range of 6–18 GHz. Figure S5 demonstrated simulated S-parameters of different transmission structures with ±10% surface conductivity variation (18 GHz@ 6.3 W).

(4) $$\sigma_{s}=\frac{1}{\rho_{0}\left[1+\alpha \left(T-T_{0}\right)\right]\left\{1+\frac{2}{\pi }\tan^{-1}\left[1.4{\left(\frac{\Delta_{\mathrm{rms}}}{\sqrt{\frac{\rho_{0}\left[1+\alpha \left(T-T_{0}\right)\right]}{\pi f\mu_{0}}}}\right)}^{2}\right]\right\}}$$

*σ_s_* is the surface conductivity of metal, ρ_0_ is the electrical resistivity of metal, α is the temperature coefficient, *f* is frequency, Δ_rms is surface roughness, μ_0_ is vacuum permeability and *T* is the actual working temperature.

Since the glass material parameters vary with temperature, temperature-dependent values were incorporated into the simulation. The temperature-dependent material parameters can be written as:

(5) $$\gamma =\gamma_{0}\left[1+\beta \left(T-T_{0}\right)\right]$$

where γ is the temperature-dependent material parameter (relative permittivity, dielectric loss tangent), γ_0_ is the material parameter at reference temperature *T*_0_, where *T*_0_ = 25° C, β is the temperature coefficient, and *T* is temperature. The glass material properties involved in this paper are listed in Table 3.

**Figure 20:**
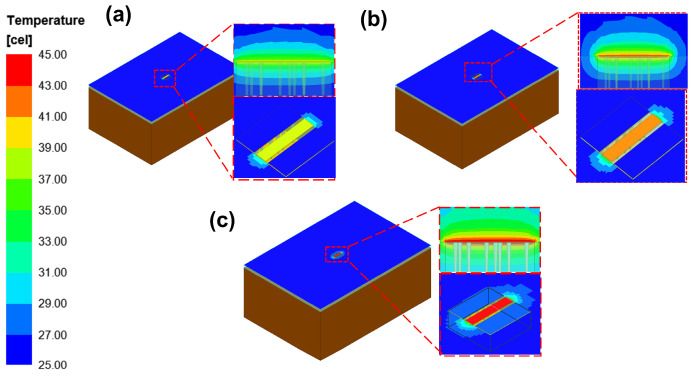
Simulated temperature rise distribution contour maps of CPW structure: (**a**) 10 W@6 GHz, (**b**) 7.9 W@12 GHz, and (**c**) 6.3 W@18 GHz.

Secondly, thermal simulation was performed in the Icepack module as shown in Figure 20. The test structure is located in the center of the sample, surrounded with other test structures (highlighted in blue). As illustrated in Figure 17b, the fabricated TGVs exhibit a tapered geometry with a top diameter of 40 μm and a bottom diameter of 10 μm. In our simulation model, the TGVs were modeled with these actual geometric dimensions to accurately represent the real operating conditions. To simplify the simulation, the surrounding test structures were modeled using the equivalent material method. The equivalent thermal conductivity was extracted using the methodology described in Ref. [35]. The computational results indicate that the equivalent thermal conductivity of the quadruple redundant TGV connected CPW test structure is 15 W/(m·K) in the XY direction and 315 W/(m·K) in the Z direction. The thermal conductivity of the brown substrate is 400 W/(m·K). The surface temperature of the brown substrate is maintained at a constant 20 °C, while the convective heat transfer coefficient for the surrounding air is set to 15 W/(m^2^·K). Radiation is neglected. A molybdenum-copper (Mo-Cu) substrate layer with dimensions consistent with the sample was positioned beneath the test structure. The thermal simulation was performed to obtain the temperature distribution. Then, temperature-dependent loss tangent and dielectric constant were updated based on the temperature distribution.

Following the material parameter updates, the electromagnetic-thermal coupled simulation proceeds iteratively until the maximum temperature differential between successive iterations falls below 0.5 °C, indicating convergence.

**Table 3:** Material property of glass [34,36].

	Relative Permittivity	Tangent Loss	Density (kg·m^−3^)	Heat Capacity (J·kg^−1^ K^−1^)
Glass_25 °C	4.18	0.0038	2.2 × 103	730
Glass_75 °C	4.20	0.0039		
Glass_175 °C	4.21	0.0044		

**Figure 21:**
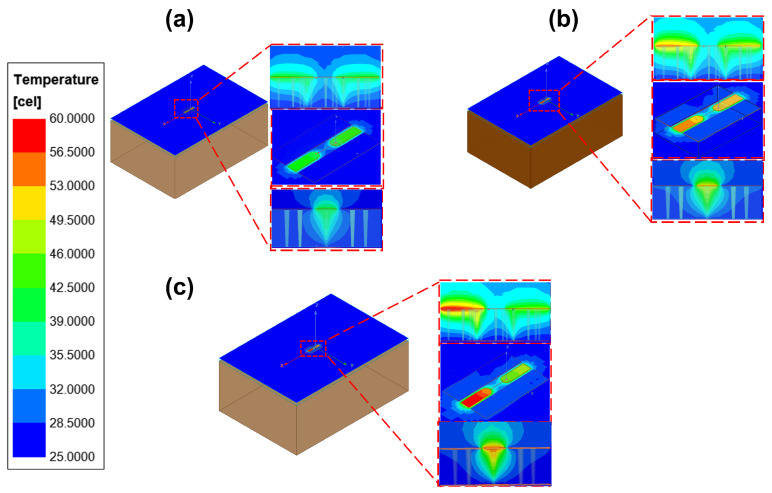
Simulated temperature rise distribution contour maps of single RF TGV connected CPW structure (**a**) 10 W@6 GHz (**b**) 7.9 W@12 GHz (**c**) 6.3 W@18 GHz.

**Figure 22:**
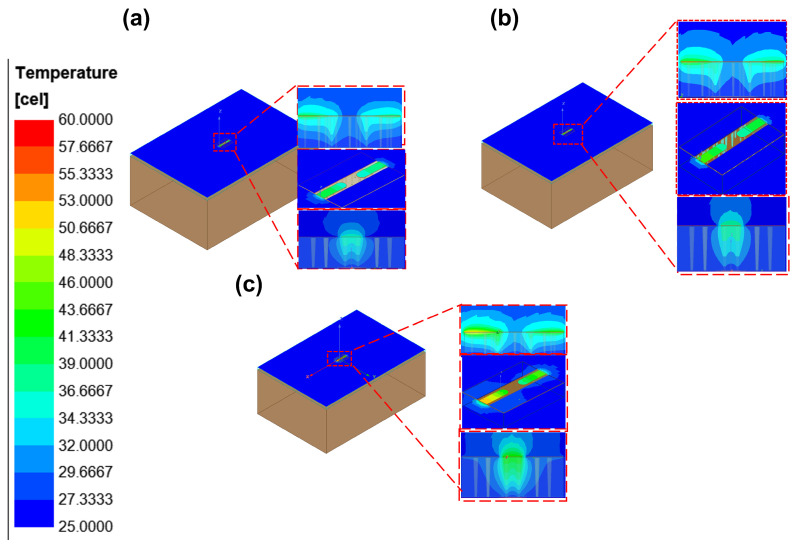
Simulated temperature rise distribution contour maps of dual-redundant RF TGV connected CPW structure (**a**) 10 W@6 GHz (**b**) 7.9 W@12 GHz (**c**) 6.3 W@18 GHz.

**Figure 23:**
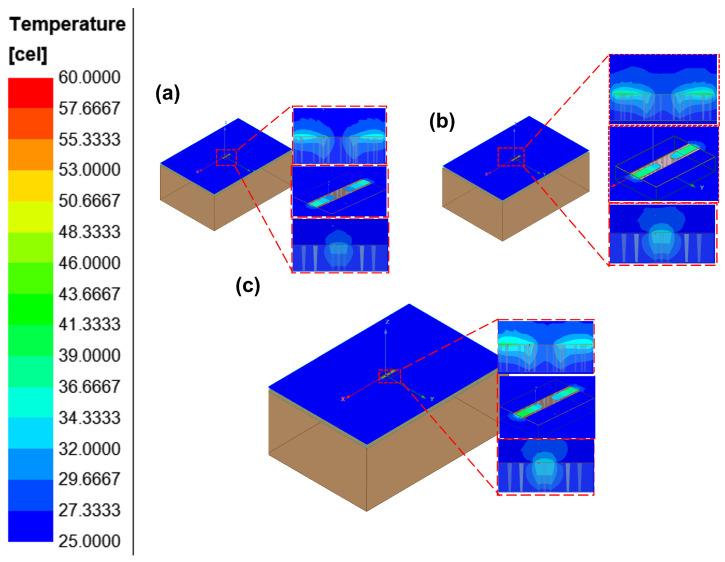
Simulated temperature rise distribution contour maps of quad-redundant RF TGV connected CPW structure (**a**) 10 W@6 GHz (**b**) 7.9 W@12 GHz (**c**) 6.3 W@18 GHz.

**Figure 24:**
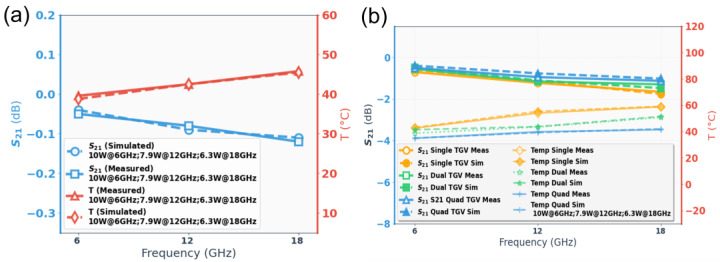
Comparison of measured and simulated S21 parameters and temperature rise in the structure versus frequency under large power signal input conditions (10 W@6 GHz, 7.9 W@12 GHz, 6.3 W@18 GHz), factoring surface roughness and electro-thermal coupling effects (**a**) CPW (**b**) RF TGV connected CPW structures.

Through the electro-thermal coupling simulation, the simulated temperature distribution contour maps for CPW, and RF TGV connected CPW structures were obtained, as shown in Figure 20, Figure 21, Figure 22 and Figure 23. The figures include magnified views of the RF signal TGV, displaying the longitudinal cross-section, top view, and transverse cross-section from top to bottom, respectively. Figure 24 presents a comparison between simulated and measured results of insertion loss and temperature rise for CPW and RF TGV connected CPW structures under large-signal conditions. The cross-sectional temperature distributions in the inset of Figure 21, Figure 22 and Figure 23 reveal that the maximum temperature occurs at the TGV surface rather than in the interior. This is primarily due to the efficient heat dissipation path formed by the close contact between the TGV bottom and the metal substrate, which enables rapid downward heat conduction through the via. This finding validates that infrared surface temperature measurements effectively represent the peak temperature of TGV structures. The temperature distribution along the Z-axis shows a characteristic gradient, with the highest temperature at the surface and decreasing temperatures toward the bottom. For single-TGV, dual-redundant TGV, and quad-redundant TGV structures, the simulated surface-to-internal maximum temperature differences are approximately 17.9 °C, 16 °C, and 10 °C, respectively.

Under large-signal conditions, the RF TGV connected CPW structure exhibits more insertion loss degradation compared to CPW structures. For the RF TGV connected CPW structure, signal transmission must traverse the RF TGV. As frequency increases, the skin depth continuously decreases. As the skin layer of the TGV sidewalls comprises a Ti/Cu composite barrier layer, where the low conductivity of Ti leads to a significant increase in surface impedance. Additionally, the high surface roughness of the TGV sidewalls further reduces the effective surface conductivity. These two factors act synergistically, causing insertion loss increase with frequency, further leading to temperature rise, the degradation of metal conductivity couples with the aforementioned high-frequency effects, further exacerbating the transmission loss degradation of the RF TGV connected CPW structure. In this study, considering the manufacturability of both LIDE and electroplating processes, we propose a quadruple-redundant RF TGV design with optimized TGV diameter, TGV pitch, and substrate thickness, which reduces the parasitic losses between vias. Through electro-thermal coupling experiments and simulation analysis, we quantitatively investigated the effects of single RF TGV, dual RF TGV, and quadruple-redundant RF TGV on S-parameters, heat flux density distribution, and temperature rise, as shown in Figure 24. The results demonstrate that the optimized quadruple-redundant via structure enables more uniform current distribution, and reduces local current density peaks and maximum heat flux density peaks, thereby effectively suppressing temperature rise under high-frequency and high-power operating conditions. Furthermore, the study reveals significant electro-thermal coupling effects under high-power and high-frequency application conditions. The quadruple-redundant via design reduces both loss and thermal resistance, resulting in decreased temperature rise. The reduced temperature, in turn, further decreases the dielectric loss and conductor loss of the transmission structure. This coupling mechanism leads to significant performance improvement in the optimized quadruple-redundant structure.

Designers should select structures based on specific application requirements. For application scenarios that do not require vertical interconnection, conventional CPW structures exhibit the lowest insertion loss with relatively simpler fabrication processes, making them the optimal choice for planar transmission. When applications require three-dimensional vertical interconnection, TGV structures should be considered. Under the condition of consistent signal line width, single-via, dual-redundant, and quadruple-redundant RF TGV structures occupy essentially the same footprint area, while the quadruple-redundant structure demonstrates superior performance in both insertion loss and thermal characteristics. Therefore, when area is not strictly constrained, the quadruple-redundant RF TGV should be prioritized to achieve optimal electro-thermal performance.

### 5.3. TGV-Based Transmission Structure Self-Heating Thermal Resistance and Temperature Rise Theoretical Analysis

RF TGV connected CPW structure demonstrates different temperature distribution characteristics, namely that the input terminal temperature is higher than the output terminal temperature, as shown in Figure 21, Figure 22 and Figure 23. In the electrical model, fully symmetrical wave ports are set at both ends of the transmission line to ensure consistency of excitation conditions. For the thermal model, fully symmetrical geometric structures are adopted at the input and output ends, with consistent boundary conditions and heat dissipation conditions (Figure 21, Figure 22 and Figure 23). Through the above symmetrical modeling, we eliminated the influence of asymmetric heat dissipation between the connectors and test fixtures. Simulation results show that under the same length condition of 1200 μm, as the number of redundant holes increases, the transmission loss gradually increases (Figure 24b). Meanwhile, the temperature difference between the input and output ends also shows a significant increasing trend, confirming that the temperature difference is generated along the direction of power attenuation in the transmission line. The magnitude of the temperature difference is mainly related to the structure, with length having a smaller influence.

#### 5.3.1. CPW Transmission Structure Self-Heating Thermal Resistance and Temperature Rise Theoretical Analysis

According to the analysis of the results shown in Figure 1 above, the electrical-thermal model was established. It was assumed that the CPW structure is uniform at both ends with perfect matching, without non-uniformity or standing wave effects. We assumed that the electromagnetic losses generated by the signal line are completely converted to thermal energy. The electromagnetic losses of the CPW transmission structure consists of two parts, dielectric loss and conductor loss. The calculation formulas are as follows [37,38].

Where *α_c_* is conductor loss coefficient, *α_d_* is the dielectric loss coefficient, and α*_cpw_* is the total loss:

*α_cpw_* = *α_c_* + *α_d_* (6)

When 1 W of power is input, the signal line loss can be represented by absorbed power *P*, which is expressed by the following formulas:

(7) $$\Delta P_{c}=1-\exp\left(-2\alpha_{\mathrm{cpw}}\right)\;W/m$$

The signal line acts as the primary distributed heat source with power density determined by the electromagnetic loss calculations, and heat conducts downward through the glass substrate to the grounded layer. In terms of heat conduction pathways, this structure can be categorized into three parallel thermal conduction regions, two side regions with ground TGVs have low thermal resistance, while the middle region without TGVs has high thermal resistance.

The total thermal resistance is calculated as three regions in parallel, with the calculation process as follows [39,40]:

(8) $$\frac{1}{R_{\mathrm{total}}}=\frac{1}{R_{\mathrm{left}}}+\frac{1}{R_{\mathrm{center}}}+\frac{1}{R_{\mathrm{right}}}$$

(9) $$\Delta T=0.2303\alpha \_cpwlP_{\mathrm{input}}R_{total}$$

Based on thermal resistance, we can obtain the temperature rise in the transmission structure, where *R_total_* is the total thermal resistance, *R_left_* is the thermal resistance of the left region, *R_center_* is the thermal resistance of the center region, *R_right_* is the thermal resistance of the right region, l is the length of the transmission line, and *P_input_* represents the input power. The detailed derivation is provided in the Supporting Information.

#### 5.3.2. TGV Self-Heating Thermal Resistance and Temperature Rise Theoretical Analysis

The analysis of Figure 21, Figure 22 and Figure 23 reveals that the temperature rise distribution pattern of the RF TGV connected CPW transmission structure differs from those of the CPW. Specifically, this structure exhibits a higher temperature rise at the input terminal compared to the output terminal. Notably, the RF TGV connected CPW transmission structure differs from the conventional CPW structure solely in the incorporation of signal TGVs. However, this modification leads to a significantly altered temperature distribution pattern. It is hypothesized that this distinctive temperature rise distribution may be attributed to the TGVs modifying the electro-thermal characteristics of the transmission structure. Therefore, electro-thermal coupling theoretical modeling and analysis were conducted to investigate the TGV structure.

**Figure 25:**
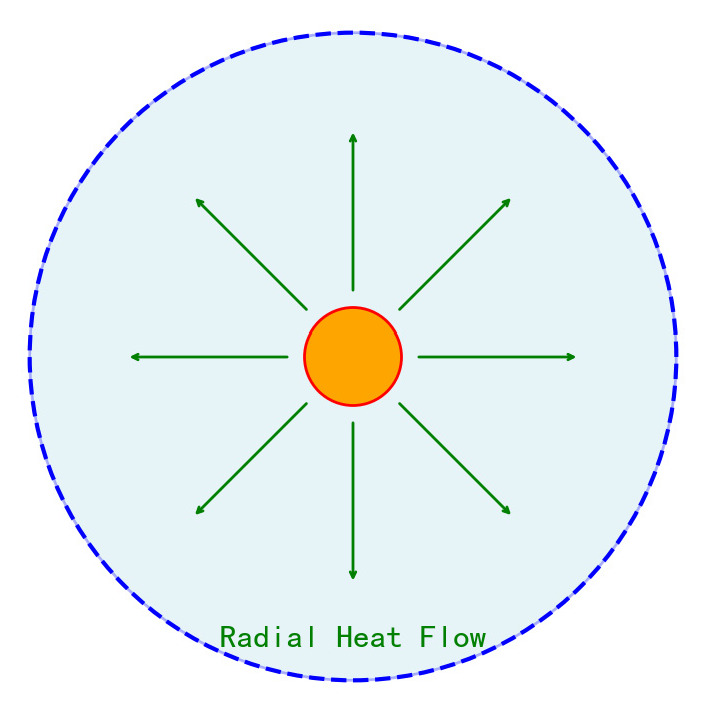
TGV electro-thermal coupling effect equivalent model.

The electro-thermal coupling modeling of TGV structures employs a coaxial structure analogy. The geometric configuration uses a cylindrical shell model where the conductive TGV sidewall serves as an equivalent heat source, creating radial heat conduction from center to periphery (Figure 25) [41]. The modeling assumes uniform transmission lines with perfect matching at both ends, neglecting non-uniformity, standing wave effects, and dielectric losses. The thermal boundary conditions specify inner conductor temperature as *T_max_* and outer conductor temperature as *T_amb_*, with adiabatic surroundings ensuring radial heat flow.

The conductor loss of the TGV can be derived as follows:

(10) $$\alpha_{c}=\frac{\sqrt{\frac{\pi f\mu_{0}}{\sigma }}}{4\pi \times Z_{0}}\left[\frac{1}{r_{tgv}}+\frac{1}{b}\right]$$

In the TGV structure designed in this work, the central signal via is surrounded by a discretely distributed array of ground vias, while the ideal coaxial model assumes a continuous cylindrical outer conductor. This geometric discrepancy indeed exists. In the TGV structure studied in this paper, the minimum distance *b* from the ground via array to the central signal via is approximately 10 times the signal via radius *r_tgv_*. Based on Equation (10), when *b/r_tgv_* ≥ 10, the conductor loss saturates with increasing *b/r_tgv_* ratio, conductor loss approaches saturation.

According to coaxial transmission line theory, the radial electric field intensity decays as follows:

(11) $$E\left(r\right)\propto \frac{1}{r}$$

At *b* = 10 *r_tgv_*, the electric field intensity at the outer boundary decreased to approximately 10% of the field strength at the inner conductor surface. Under this condition, the geometric perturbation of the outer conductor boundary (such as the discontinuity introduced by the discrete ground via array) has a negligible impact on the field distribution. Based on the above analysis, we conclude that the validity boundary condition for the coaxial approximation is that the distance between the ground vias and the central signal via should be greater than 10 times the signal via radius.

Based on the principle of thermal equilibrium, the losses generated by the central TGV are converted into heat. Heat flow is distributed radially, propagating from the center outward. Based on steady-state heat conduction conditions and Fourier’s law of heat conduction [23,42]:

(12) $$R_{tgv}=\frac{\Delta T}{Q}=\frac{1}{k\cdot 2\pi L}\ln\left(\frac{b}{r_{tgv}}\right)$$

where *r* is the direction along the heat flow, *L* is the substrate thickness, *q* is the heat flux density, *Q* is the heat quantity, and *k* is the thermal conductivity.

(13) $$\Delta T=-\frac{P_{loss}}{k\cdot 2\pi L}\ln\left(\frac{b}{r_{tgv}}\right)$$

where Δ*T* is the temperature difference, *R_tgv_* is the thermal resistance, k is the material thermal conductivity, *P_loss_* is the thermal dissipation power, P_input_ is the input power, *T_max_* is the maximum temperature at the TGV sidewall, and *T_amp_* is the ambient temperature. The detailed derivation is provided in the Supporting Information.

**Figure 26:**
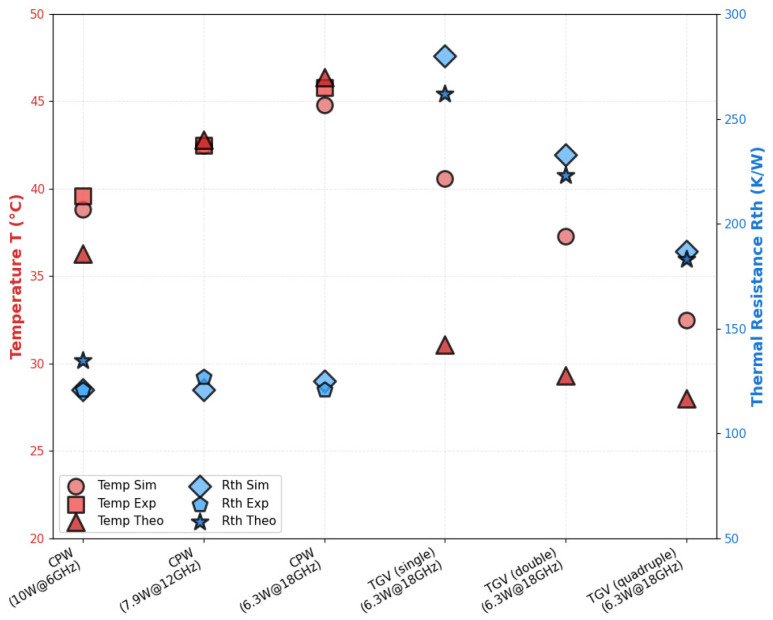
Comparison of simulation (sim)-experimental (exp)-theoretical (theo) results for temperature rise and thermal resistance of different transmission structures.

Based on the theoretical derivation presented above, Table S1 summarizes the transmission loss coefficients of the CPW, and TGV connected CPW structures, while Figure 26 presents a comparison of the experimental, simulation, and theoretical results for temperature rise and thermal resistance of different transmission structures.

When high-power RF signals propagate through the transmission structure, attenuation occurs along the transmission direction. This attenuation results in lower power dissipation at the output port relative to the input port of the transmission structure. Therefore, for symmetric structures with identical thermal resistance on both sides, the output port exhibits a lower temperature. As shown in Table S1, the CPW structure, due to its lower transmission loss, exhibits similar heat dissipation power at both the output and input ports. Combined with its lower thermal resistance (as shown in Figure 26), this ultimately results in a slightly lower temperature at the output port compared to the input port. For the single RF TGV connected CPW transmission structure, both the transmission loss and thermal resistance are relatively high, resulting in a significantly higher temperature at the input port compared to the output port.

### 5.4. Performance of TGV Transmission Structures Under Large-Signal Continuous Wave (20 W) and Pulsed Wave Conditions (100 W)

**Figure 27:**
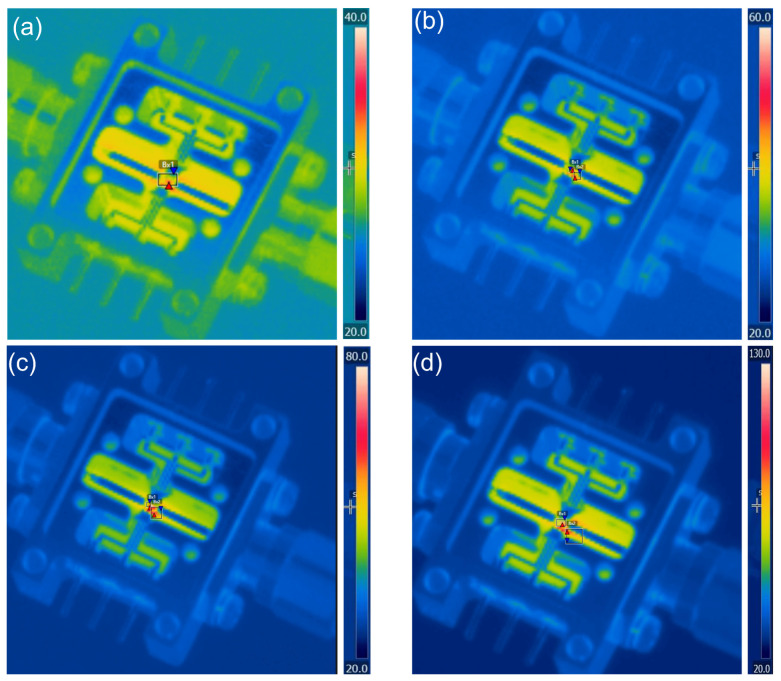
Measured thermal images of RF TGV connected CPW structure under different input powers: (**a**) 5 W (**b**) 10 W (**c**) 15 W (**d**) 20 W.

**Figure 28:**
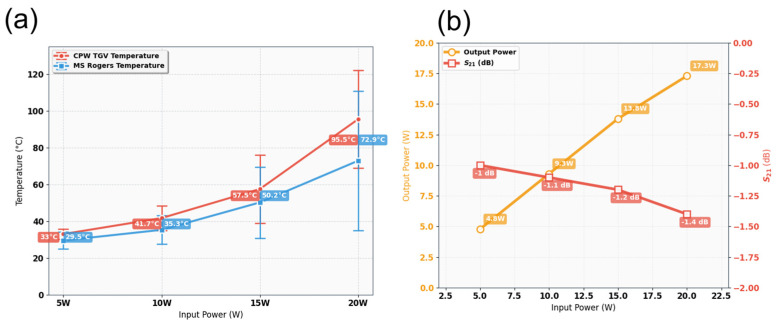
(**a**) The maximum temperature rise in microstrip and RF TGV connected CPW structure. (**b**) Variation in insertion loss and output power of CPW.

According to the findings from Section 5.2, the quad-redundant RF TGV connected CPW structure demonstrates enhanced electrical performance under high-power signal conditions, while enabling through-layer interconnection through signal TGV implementation. Here, the designed quad-redundant RF TGV connected CPW structure is chosen to be assembled in the test fixture, which is measuring in 1200 μm CPW in length. The temperature field distribution and insertion loss of RF TGV Connected CPW structures and microstrip lines are extracted. In Figure 27 measured thermal images are shown of RF TGV connected CPW structure under different input powers. The extracted temperature as the CW RF signal input ranges from 5 W to 20 W is summarized in Figure 28.

It was found that when input power is gradually increased from 5 W to 20 W, the maximum temperature changes from 36.1 °C to 128.5 °C as shown in Figure 28a. In contrast, the microstrip line structure based on Rogers 5800 substrate increases from 34 °C to 115.5 °C, with the temperature difference between the two structures maintained within a range of 2.1 °C to 13 °C. With RF signal transmission, the RF TGV connected CPW structure exhibits a maximum temperature rise 12.5% higher than that of Rogers 5800, but its width is only 1/3 of the microstrip lines on Rogers 5800. Using the electro-thermal coupling simulation approach described in Section 5.2, electro-thermal coupling simulations were performed for the RF TGV connected CPW structure (Figure S6). As shown in Figure 28b, when input power is gradually increased from 5 W to 20 W, the insertion loss of RF TGV connected CPW structure rises from 1 dB to 1.4 dB. This trend correlates with increased conductor losses and dielectric losses caused by material thermal effects; however, the maximum insertion loss degradation is only 0.4 dB. These test results indicate a good linear response relationship between output power and input power, demonstrating that this structure exhibits good electrical performance under high-power conditions.

In practical RF applications, transmission structures handle not only continuous wave but also pulsed wave power signals depending on specific system requirements. In pulsed mode, the transmitter transmits signals for brief periods before shutting down, followed by receiver activation to detect return echoes. This switching mechanism conserves power and mitigates heat generation compared to continuous operation. To evaluate the structure’s versatility, this study investigates the performance of the 1200 μm quadruple RF TGV connected CPW structures under the test conditions of 500 μs period, 12.5 μs pulse width, 2.5% duty cycle, and input power ranging from 25 W to 100 W.

**Figure 29:**
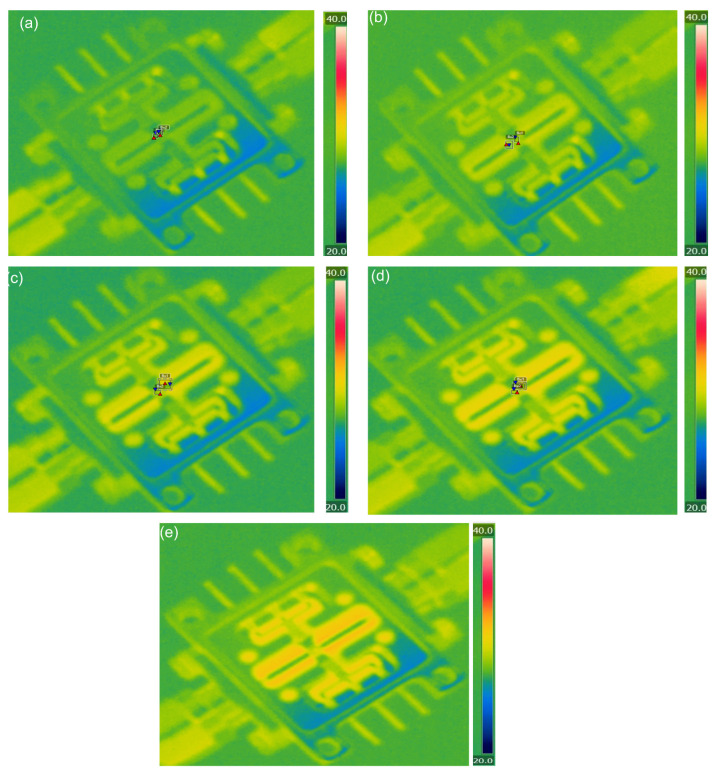
Temperature rise contour maps of RF TGV connected CPW structure under (**a**) 25 W, (**b**) 50 W, (**c**) 60 W, (**d**) 80 W, and (**e**) 100 W pulsed wave conditions.

**Figure 30:**
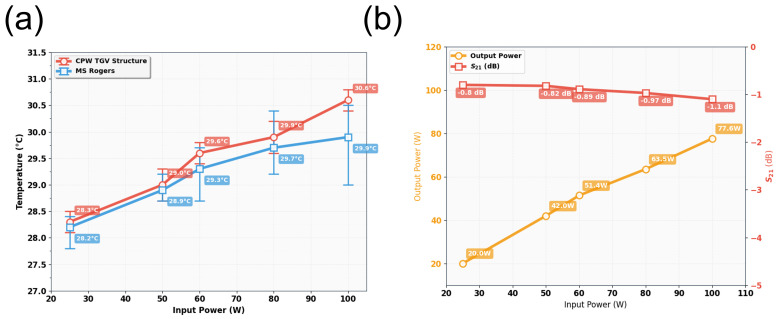
(**a**) Comparative analysis of maximum temperature rise variation with input pulsed wave signal for RF TGV connected CPW structure and Rogers microstrip line. (**b**) Variation in output power and S_21_ parameters with input power for 1200 μm RF TGV connected CPW under pulsed wave conditions.

Figure 29 presents the temperature rise contour maps of RF TGV connected CPW structures under 25 W, 50 W, 60 W, 80 W, and 100 W pulsed wave conditions. Based on the data from Figure 29, we plotted the maximum temperature rise variation with input pulsed wave signal for both the RF TGV connected CPW structure and Rogers microstrip lines, as shown in Figure 30. It can be seen that when the input power is gradually increased from 25 W to 100 W, the maximum temperature changes from 28.5 °C to 30.6 °C, which is comparable to the temperature of microstrip lines on the Rogers 5800. The temperature rise under the pulsed operation mode is lower than that under the continuous wave mode. This phenomenon is primarily attributed to the effective reduction in average power density in the structure by pulsed signals.

Meanwhile, insertion loss remains relatively stable throughout the entire test power range, with S_21_ parameters varying from 0.82 dB to 1.0 dB, representing a variation of only approximately 0.2 dB. The output power varies linearly from 20 W to 77 W for input powers of 25 W to 100 W, as shown in Figure 30. This demonstrates that the structure maintained low insertion loss even under high-power operating conditions.

**Figure 31:**
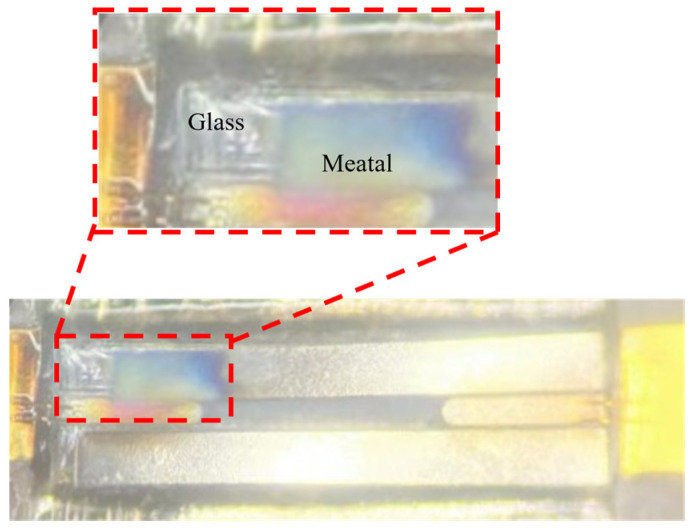
Microscopic observation of metal-glass interface delamination.

If power were to be increased further, dielectric loss and conductor loss would be the primary mechanisms triggering rapid performance degradation. As power increases, the temperature rises, leading to significant increases in both dielectric and conductor losses, which would result in rapid degradation of transmission performance. In contrast, metal electromigration is a relatively slow cumulative process and would not cause rapid performance degradation. During the power tests, when the input power exceeded these thresholds, thermal-induced failure of the TGV structure was observed. As shown in the red dashed box in Figure 31, the metal layer at the input port in the upper left corner exhibited burning damage, and delamination occurred between the metal trace and the glass substrate. This failure mechanism is primarily attributed to the sharp temperature rise under high-power conditions. The significant coefficient of thermal expansion (CTE) mismatch between the metal and glass generates substantial thermal stress at the interface. When this thermal stress exceeds the interfacial bonding strength, it triggers metal delamination, ultimately leading to structural failure and loss of electrical conductivity.

Table 4 compares the research status of TSV and TGV technologies in high-frequency applications. Current research demonstrates that TGV-based transmission structures outperform TSV architectures in high-frequency applications. However, despite substantial progress in small-signal characterization, a critical knowledge gap persists in the current research landscape. Existing studies on both glass-based TGV structures and silicon-based TSV configurations predominantly focus on low-power scenarios, leaving high-power, performance largely unexplored. Moreover, experimental investigations concerning electro-thermal coupling effects remain largely unexplored. This work conducted electro-thermal characterization of CPW and RF TGV connected CPW structures under high-power conditions. The quadruple redundant RF TGV connected CPW structure demonstrates superior thermal management and maintains low insertion loss performance compared to single and double redundant configurations.

## 6. Conclusions

This work presents an RF TGV transmission structure design incorporating optimized ground via layout and quadruple-redundant signal structure, and fabricates a high-frequency high-power transmission structure. Through the mitigation of local current density peaks and heat flux density peaks, the structure achieves concurrent reductions in insertion loss, thermal resistance, and temperature rise, exhibiting reliable high-power transmission capability under both 20 W continuous wave and 100 W pulsed wave operating conditions as validated through experimental characterization. At 6.3 W@18 GHz, the single, double, and quadruple redundant RF TGV connected CPW structures exhibited insertion losses of 1.63 dB, 1.30 dB, and 1.12 dB, with corresponding temperature rises of 58.8 °C, 50.8 °C, and 41.4 °C, respectively. The results demonstrate that high-power signals lead to increased insertion loss and temperature rise, while increasing via redundancy reduces both loss and temperature. This can be attributed to the mechanism where the optimized quadruple-redundant design with ground via layout optimization reduces local current density peaks and heat flux density peaks, thereby simultaneously lowering thermal resistance and temperature rise. The loss degradation mechanism under high-frequency, high-power conditions was elucidated. TGV structures exhibit lower surface conductivity than CPW structures because sidewall corrugation extends the current path and the titanium outer layer has lower conductivity. At high frequencies, the skin effect confines the current to the surface where low-conductivity titanium impedes transport, significantly reducing effective conductivity. Coupled with thermal effects, surface conductivity deteriorates further, increasing insertion loss. Comparative analysis of attenuation coefficients and thermal resistance characteristics across different structures revealed that signal attenuation along the transmission path causes the input port to exhibit lower temperature rise than the output port. Based on the analysis and correlations between experimental and simulation results, we confirmed the electrothermal coupling mechanism whereby surface conductivity degradation induces increased loss and temperature rise as the designed TGV RF transmission structures transmit high power high frequency signals. A simulation scheme considering the electrothermal coupling effects was established as a design methodology to implement the design of high-power high-frequency TGV based RF transmission structures. For application-oriented validation, the optimized quadruple redundant RF TGV connected CPW was mounted in fixtures simulating real operating conditions. The impact of power increase on structural performance exhibits significant operating mode dependence. Under continuous wave conditions, as power increased from 5 W to 20 W, temperature rose from 36.1 °C to 128.5 °C, while insertion loss showed low nonlinearity, only increasing from 1 dB to 1.4 dB—less than proportional to power increase. Under pulsed wave operation (2.5% duty cycle), as power increased from 25 W to 100 W, temperature only rose from 28.5 °C to 30.6 °C, and insertion loss only increased from 0.8 dB to 1.1 dB. This demonstrates that the structure maintains low temperature and low insertion loss even under high-power conditions. The cooling time provided by pulse intervals effectively suppresses thermal accumulation. For high-power applications, pulsed modulation schemes can control temperature rise at low levels with minimal sacrifice in electrical performance. Therefore, designers should select appropriate power margins based on actual operating modes. Continuous wave applications are dominated by temperature rise constraints, while pulsed wave applications can prioritize electrical performance optimization.

## Figures and Tables

**Table 1:** Design parameters of the TGV based transmission structure.

Structure	Parameters	Value
CPW	H	250 μm
	T_copper	20 μm
	W	830 μm
	L_CPW	400/800 μm/1600 μm
	W_gap	40 μm
	W_signal	150 μm
RF TGV connected CPW		
	TGV diameter	40 μm
	L_1	400 μm

**Table 2:** The insertion loss characteristics of probes and cables at various excitation states.

Frequency@power	Probe Loss (dBm)	Cable Loss (dBm)
6 GHz@10 W	0.178	1.17
12 GHz@7.9 W	0.247	1.69
18 GHz@6.3 W	0.369	1.95

**Table 4:** Research status of TSV and TGV technologies in high-frequency applications.

Reference	Material	Structure	Frequency	Insertion Loss	High-Power Signal	High-Power Insertion Loss	Maximum Temperature Rise	Thermal Resistance
[5]	ABF/Glass/ABF	CPW	20–77 GHz	0.095 dB/mm@18 GHz	/	/	/	
		MS		0.13 dB/mm@18 GHz	/	/	/	
[20]	Glass	TGV	DC-80 GHz	0.558 dB@18 GHz (single)	/	/	/	
		CPW		0.062 dB/mm@18 GHz	/	/		
[30]	Si	TSV	DC-60 GHz	0.22 dB@18 GHz (single)	/	/	/	
[43]	Si	MS	DC-50 GHz	0.4 dB/mm@18 GHz	5 W @18 GHz	/	150 °C	341 K/W
[44]	Si	CPW	DC-50 GHz	1.2 dB/mm@18 GHz	/	/	200 °C	
[25]	SiON	CPW	DC-50 GHz	2.63 dB/mm@18 GHz	10 W @18 GHz	/	150 °C	29.1 K/W
This work	Glass	CPW	DC-40 GHz	0.093 dB/mm@18 GHz	6.3 W	0.12 dB/mm @18 GHz	45.8 °C	121 K/W
		Single_TGV	DC-40 GHz	0.776 dB@18 GHz_1.2 mm	6.3 W	1.6 dB@18 GHz_1.2 mm	58.8 °C	280 K/W (single TGV)
		Double_TGV		0.432 dB @18 GHz_1.2 mm	6.3 W	1.3 dB@18 GHz_1.2 mm	50.8 °C	233 K/W (Double_TGV)
		Quadruple_TGV		0.312 dB@18 GHz_1.2 mm	6.3 W	1.1 dB@18 GHz_1.2 mm	41.4 °C (compared to 0.3 dB degradation at 25 °C)	187 K/W (quadruple_TGV)
				/	20 W @20 GHz (CW)	1.4 dB@20 GHz_20 W_1.2 mm (CW)	128.5 °C (compared to 0.4 dB degradation at 25 °C)	
				/	100 W @20 GHz (PW)	1.1 dB @20 GHz_100 W_1.2 mm (PW)	31.3 °C (compared to 0.3 dB degradation at 25 °C)	

## Data Availability

The data presented in this study are available on request from the corresponding author. The data are not publicly available due to privacy.

## Supplementary Materials

The following supporting information can be downloaded at: https://www.mdpi.com/article/10.3390/mi17020253/s1, Figure S1: Simulated heat flux density distribution of CPW structures under 1 W@18 GHz conditions (a) 400 μm (b) 800 μm (c) 1600 μm. Figure S2: Temperature rise distribution of CPW structures under 1 W@18 GHz conditions (a) 400 μm (b) 800 μm (c) 1600 μm. Figure S3: 3D full-wave simulation models of RF TGV connected CPWs with different configurations (a)single TGV, (b)dual-redundant TGV, and (c)quad-redundant TGV transmission structures. Figure S4: Comparison of simulated and measured S21 parameters for different structures at 18GHz@1mW. Figure S5: Simulated S-parameters of different transmission structures with ±10% surface conductivity variation (18 GHz @ 6.3 W). Figure S6: Simulated thermal images of RF TGV connected CPW structure under different input powers: (a) 5W (b) 10W (c) 15W (d) 20W. Table S1: Transmission loss coefficient of CPW and TGV connected CPW structures.
