# Supplementary material for: Electro-Thermal Co-Design and Verification of TGV Transmission Structures for High-Power High-Frequency Applications
Source: Micromachines (Basel). 2026 Feb 16;17(2):253. doi: 10.3390/mi17020253 (PMC12942756; doi:10.3390/mi17020253)
Supplement: Supplementary file 1 [file micromachines-17-00253-s001.zip › micromachines-4108766-supplementary.pdf]

Support Information

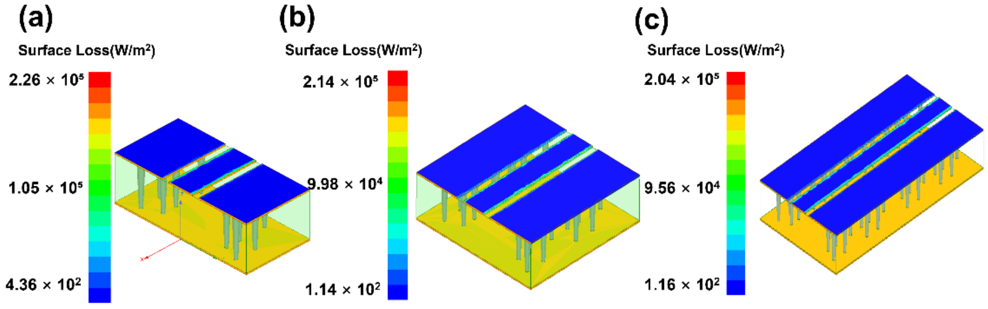

Figure S1. Simulated heat flux density distribution of CPW structures under 1 W@18 GHz conditions (a) 400 μm (b) 800 μm (c) 1600 μm

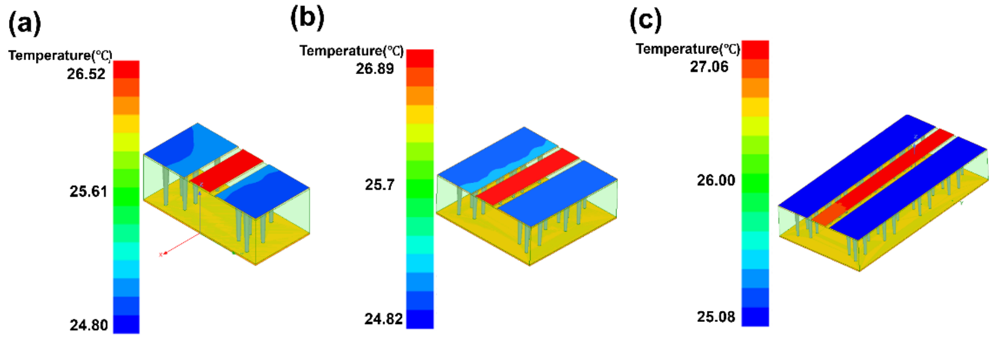

Figure S2. Temperature rise distribution of CPW structures under 1 W@18 GHz conditions (a) 400 μm (b) 800 μm (c) 1600 μm

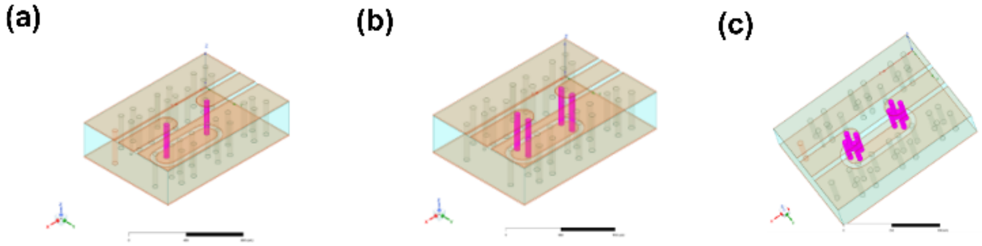

Figure S3. 3D full-wave simulation models of RF TGV connected CPWs with different configurations (a)single TGV, (b)dual-redundant TGV, and(c)quad-redundant TGV transmission structures

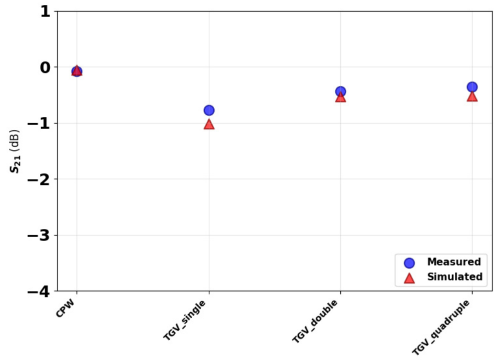

Figure S4. Comparison of simulated and measured S21 parameters for different structures at 18GHz@1mW

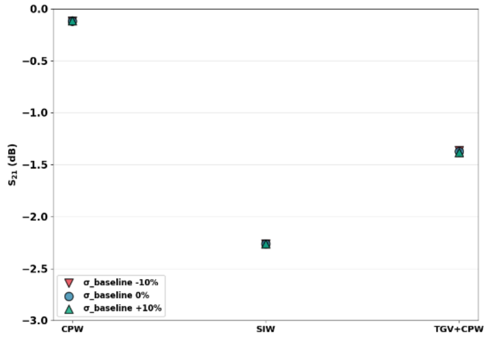

Figure S5. Simulated S-parameters of different transmission structures with ±10% surface conductivity variation (18 GHz @ 6.3 W)

CPW Transmission Structure Self-Heating Thermal Resistance and Temperature Rise Theoretical Analysis

The electrical-thermal model is established. It is assumed that the CPW structure is uniform at both ends with perfect matching, without non-uniformity or standing wave effects. We assume that the electromagnetic losses generated by the signal line are completely converted to thermal energy. The electromagnetic losses of the CPW transmission structure consists of two parts, dielectric loss and conductor loss. The calculation formulas are as follows [1]

$$\alpha_d = 27.3 \cdot \frac{\varepsilon_r}{(\varepsilon_{eff})^{0.5}} \cdot \frac{\varepsilon_{eff}-1}{\varepsilon_r-1} \cdot \frac{\tan\delta}{\lambda_0}\,\text{dB/cm} \tag{S1}$$

$$\varepsilon_{eff} = \frac{\varepsilon_r+1}{2} + \frac{\varepsilon_r-1}{2} \cdot \frac{1}{\sqrt{1+12h/W}} \tag{S2}$$

$$\alpha_c = \frac{8.68}{Z_0 W}\sqrt{\frac{\pi f \mu_0}{\sigma}}\ \text{dB/cm} \tag{S3}$$

Where α_c is conductor loss coefficient, α_d is the dielectric loss coefficient, and α is the total loss:

$$\alpha\_{cpw} = \alpha_c + \alpha_d \tag{S4}$$

Where ε_r is the relative permittivity, ε_{eff} is the effective relative permittivity, σ is the conductivity, λ_0 is the wavelength in vacuum, tan δ represents the dielectric loss tangent, h represents the substrate height, w represents the signal line width, Z_0 represents the impedance of 50 Ω, and f represents the transmission frequency[2].

When 1 W of power is input, the signal line loss can be represented by absorbed power P, which is expressed by the following formulas:

$$P = \exp(-2\alpha\_cpw) \tag{S5}$$

$$\Delta P_c = 1 - \exp(-2\alpha\_cpw\,)\ \text{W/m} \tag{S6}$$
$$= 0.2303\alpha\ \text{W/m} \tag{S7}$$

In order to establish the thermal model with the calculated electromagnetic losses as heat sources, several boundary conditions are applied, radiation heat transfer is neglected, the ground plane is maintained at a constant temperature of 25 °C, and surrounding edges are treated as adiabatic boundaries. Under these conditions, the signal line acts as the primary distributed heat source with power density determined by the electromagnetic loss calculations, and heat conducts downward through the glass substrate to the grounded layer. In terms of heat conduction pathways, this structure can be categorized into three parallel thermal conduction regions, two side regions with ground TGVs have low thermal resistance, while the middle region without TGVs has high thermal resistance. The thermal resistance of the middle region can be calculated by the following formula. Taking a uniform signal line of size 2a×2b as a uniform heat source, with a glass substrate of size 2c×2d and thickness t, substrate thermal conductivity k_1, and bottom convective heat transfer coefficient h_{equ}. A direct calculation scheme is provided based on analytical formulas. The spreading thermal resistance calculation formula for this structure is [3] [4]:

$$R_{center} = \frac{c^2}{2k_1a^2d}\sum_{m=1}^{\infty}\frac{\sin^2(m\pi a/c)}{(m\pi)^3}\cdot\phi(\delta_m)$$
$$+\frac{d^2}{2k_1b^2c}\sum_{n=1}^{\infty}\frac{\sin^2(n\pi b/d)}{(n\pi)^3}\cdot\phi(\lambda_n)$$
$$+\frac{cd}{k_1a^2b^2}\sum_{m=1}^{\infty}\sum_{n=1}^{\infty}\frac{\sin^2(m\pi a/c)\sin^2(n\pi b/d)}{(m\pi)^2(n\pi)^2\beta_{mn}}\cdot\phi(\beta_{mn}) \tag{S8}$$
$$\phi(\zeta) = \frac{e^{2\zeta t_1}+qe^{4\zeta t_1}}{e^{2\zeta t_1}+qe^{4\zeta t_1}} \tag{S9}$$
$$q = \frac{\zeta t+ht/k_1}{\zeta t-ht/k_1} \tag{S10}$$
$$\delta_m = m\pi/c \tag{S11}$$
$$\lambda_n = n\pi/d \tag{S12}$$
$$\beta_{mn} = \sqrt{(\delta_m^2+\lambda_n^2)} \tag{S13}$$

The thermal resistance of the TGV regions on both sides is calculated by the following formula:

$$R_{\text{side}} = \frac{L}{k_{\text{eff}}\times A_{\text{side}}} \tag{S14}$$

The calculation method for effective thermal conductivity is as follows, where φ may represent the volume fraction of metal

$$k_{\text{eff}} = k_{\text{glass}}\times(1-\varphi)+k_{\text{metal}}\times\varphi \tag{S15}$$

The total thermal resistance is calculated as three regions in parallel, with the calculation process as follows:

$$\frac{1}{R_{\text{total}}} = \frac{1}{R_{\text{left}}}+\frac{1}{R_{\text{center}}}+\frac{1}{R_{\text{right}}} \tag{S16}$$

Based on thermal resistance, we can obtain the temperature rise of the transmission structure, where P_{input} represent the input power.

$$\Delta T = \quad 0.2303\alpha lP_{input}R_{total} \tag{S17}$$

4.3.2 TGV Self-heating Thermal Resistance and Temperature Rise Theoretical Analysis

The electro-thermal coupling modeling of TGV structures employs a coaxial structure analogy. The geometric configuration uses a cylindrical shell model where the conductive TGV

sidewall serves as an equivalent heat source, creating radial heat conduction from center to periphery The modeling assumes uniform transmission lines with perfect matching at both ends, neglecting non-uniformity, standing wave effects, and dielectric losses. The thermal boundary conditions specify inner conductor temperature as Tmax and outer conductor temperature as Tamb, with adiabatic surroundings ensuring radial heat flow.

The conductor loss of the TGV can be derived as follows:

$$\alpha_c = \frac{\sqrt{\frac{\pi f \mu_0}{\sigma}}}{4\pi \times Z_0}\left[\frac{1}{r_{tgv}} + \frac{1}{b}\right] \quad (S18)$$

The impedance of the transmitting TGV is Z_0:

$$Z_o = 60\sqrt{\frac{\mu_r}{\varepsilon_{r\ (f)}}}\ln\left(\frac{b}{r_{tgv}}\right) \quad (S19)$$

Where μ_r is the relative permeability, μ_0 is the permeability of free space, and ε_r is the relative permittivity r_{tgv} is the radius of the TGV, b represents the equivalent outer conductor radius, i.e., at a distance of 10 times the inner conductor radius, the electromagnetic field strength has attenuated to negligible levels.

Based on the principle of thermal equilibrium, the losses generated by the central TGV are converted into heat. Heat flow is distributed radially, propagating from the center outward. Based on steady-state heat conduction conditions and Fourier's law of heat conduction: [5] [6]

$$q = k\frac{dT}{d_r} \quad (S20)$$

$$Q = -k \cdot 2\pi r L\frac{dT}{dr} \quad (S21)$$

Integration along the heat flow direction yields:

$$Q\int_{r_{tgv}}^{b}\frac{Q \cdot dr}{k \cdot 2\pi r L} = \int_{T_1}^{T_2} dT \quad (S22)$$

$$\frac{Q}{k \cdot 2\pi L}\int_{r_{tgv}}^{b}\frac{dr}{r} = \Delta T \quad (S23)$$

$$R_{tgv} = \frac{\Delta T}{Q} = \frac{1}{k \cdot 2\pi L}\ln\left(\frac{b}{r_{tgv}}\right) \quad (S24)$$

Where r is the direction along the heat flow, L is the substrate thickness, q is the heat flux density, Q is the heat quantity, and k is the thermal conductivity.

The lost heat Q equals the thermal dissipation power Ploss of the TGV:

$$P_{loss} = Q = 2P_{input}\alpha_{ctgv}L \quad (S25)$$

$$\Delta T = -\frac{P_{loss}}{k \cdot 2\pi L}\ln\left(\frac{b}{r_{tgv}}\right) \quad (S26)$$

$$T_{max} = T_{amb} + \Delta T \quad (S27)$$

Where ΔT is the temperature difference, Rtgv is the thermal resistance, k is the material thermal conductivity, P_{loss} is the thermal dissipation power, P_{input} is the input power, T_{max} is the maximum temperature at the TGV sidewall, and T_{amp} is the ambient temperature.

Table S1. Transmission loss coefficient of CPW and TGV connected CPW structures.

Transmission loss coefficient	Insertion loss（dB/mm）
α_{cpw_6GHZ}	0.045
α_{cpw_12GHZ}	0.088
α_{cpw_18GHZ}	0.141
α_{tgv_6GHZ}（single）	0.114
α_{tgv_12GHZ}（single）	0.160
α_{tgv_18GHZ}（single）	0.190
α_{tgv_6GHZ}（double）	0.081
α_{tgv_12GHZ}（double）	0.115
α_{tgv_18GHZ}（double）	0.141
α_{tgv_6GHZ}（quadruple）	0.057
α_{tgv_12GHZ}（quadruple）	0.081

α_{tgv_18GHZ} (quadruple)	0.099

[1] Note: Since experimental measurements can only capture surface temperatures of the sample, direct acquisition of actual temperature rise data inside the TGV is not feasible. The TGV data listed in the table represents the temperature rise and thermal resistance characteristics of quad-redundant TGV based on theoretical calculations and simulations.

As shown in Figure S6, the simulation results demonstrate good agreement with the experimental measurements, which confirms the accuracy of the results. These results validate the accuracy of the electro-thermal coupling design methodology presented in Sections 4.2, demonstrating that the structure exhibits excellent thermal performance under high-power signal conditions.

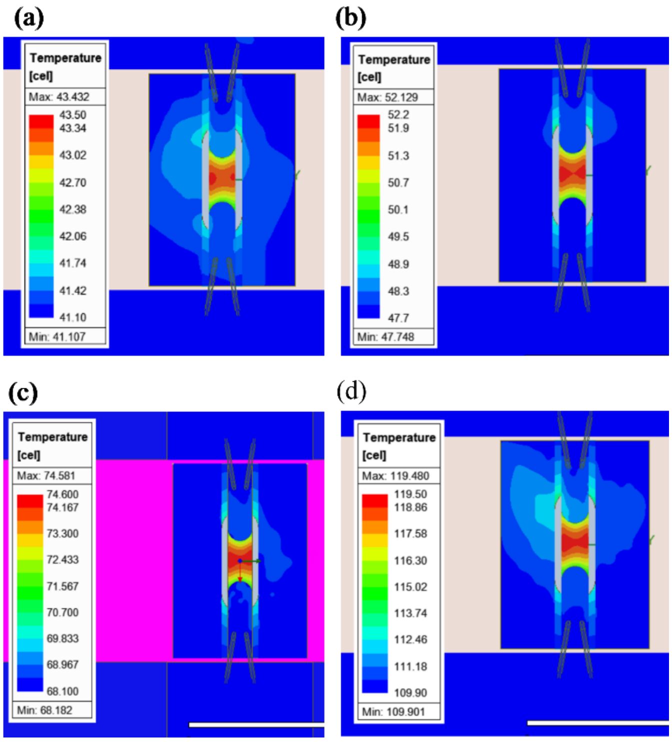

Figure S6. Simulated thermal images of RF TGV connected CPW structure under different input powers: (a) 5W (b) 10W (c) 15W (d) 20W

1. Collin, R.E. *Foundations for microwave engineering*; John Wiley & Sons: 2007.
2. Bahl, I.J. Average power handling capability of multilayer microstrip lines. *International Journal of RF and Microwave Computer‐Aided Engineering: Co‐sponsored by the Center for Advanced Manufacturing and Packaging of Microwave, Optical, and Digital Electronics (CAMPmode) at the University of Colorado at Boulder* **2001**, *11*, 385-395.
3. Muzychka, Y.S.; Yovanovich, M.M.; Culham, J.R. Influence of Geometry and

Edge Cooling on Thermal Spreading Resistance. *J. Thermophys Heat Transfer* **2006**, *20*, 247-255, doi:10.2514/1.14807.
4. Muzychka, Y.; Culham, J.; Yovanovich, M. Thermal spreading resistance of eccentric heat sources on rectangular flux channels. *J. Electron. Packag.* **2003**, *125*, 178-185.
5. Min, Q.; Li, E.-P.; Jin, J.-M.; Chen, W. Electrical–Thermal Cosimulation of Coaxial TSVs With Temperature-Dependent MOS Effect Using Equivalent Circuit Models. *IEEE Trans. Electromagn. Compat.* **2020**, *62*, 2247-2256, doi:10.1109/temc.2020.2973811.
6. Gan, H.; Xu, A.; Zhang, J. Investigation on Joule Heating of Plated Through Hole (PTH) Via. In Proceedings of the 2025 IEEE 75th Electronic Components and Technology Conference (ECTC), 2025; pp. 642-647.
